# At-risk Internet addiction and related factors among junior high school teachers—based on a nationwide cross-sectional study in Japan

**DOI:** 10.1186/s12199-018-0759-3

**Published:** 2019-01-05

**Authors:** Ayumi Iwaibara, Mari Fukuda, Hideki Tsumura, Hideyuki Kanda

**Affiliations:** 1 Department of Nursing, Faculty of Nursing and Nutrition, The University of Shimane, 151 Nishihayashigi-cho, Izumo, Shimane 693-8550 Japan; 2 Department of Environmental Medicine and Public Health, Faculty of Medicine, Shimane University, 89-1, Enya-cho, Izumo, Shimane 693-8501 Japan; 3 Department of Psychology, Faculty of Integrated Arts and Sciences, Tokushima University, 2-1, Minamijyouzanjima-cho, Tokushima, 770-8502 Japan

**Keywords:** Internet addiction, Burnout syndrome, Junior high school teacher, Nationwide survey

## Abstract

**Background:**

School teachers have a possibility toward at-risk Internet addiction (IA) due to increased opportunities to use the Internet, along with the spread of the Internet in recent years. Burnout syndrome (BOS) is found to be one of the symptoms related to unhealthy mental health, especially among teachers. This study aims to research the relationship between at-risk IA and the Internet usage or BOS by conducting a nationwide cross-sectional survey and examining the factors associated with IA.

**Method:**

This study was a cross-sectional survey by anonymous questionnaire. This survey was a random sampling survey of junior high schools across Japan in 2016. The participants were 1696 teachers at 73 schools (response rate in teachers 51.0%). We asked participants for details of their backgrounds, Internet usage, the Internet Addiction Test (IAT) by Young, and the Japanese Burnout Scale (JBS). We divided the participants into either the at-risk IA group (IAT score ≧ 40, *n* = 96) or the non-IA group (IAT score < 40, *n* = 1600). To compare the difference between at-risk IA and non-IA, we used nonparametric tests and *t* test according to variables. To analyze the relationship between the IAT score and the scores of three factors of the JBS (emotional exhaustion, depersonalization, and personal accomplishment), we used both ANOVA and ANCOVA, adjusted by relevant confounding factors. To clarify the contribution of each independent variable to IAT scores, we used multiple logistic regression analysis.

**Results:**

In our study, at-risk IA was associated with using the internet many hours privately, being on the Internet both on weekdays and weekends, playing games, and surfing the Internet. In the relationship between IAT score and BOS factor score, a higher score for “depersonalization” had a positive relationship with at-risk IA, and the highest quartile for “decline of personal accomplishment” had a lower odds ratio with at-risk IA by multiple logistic regression analysis.

**Conclusion:**

We clarified there is a significant relationship between at-risk IA and BOS among junior high school teachers in a nationwide survey. Our results suggest that finding depersonalization at the early stage may lead to the prevention of at-risk IA among teachers. Those who are at-risk of IA may feel personal accomplishment through use of the Internet.

## Background

Internet addiction (IA) has increased with the rapid spread of Internet use in recent years. According to a survey by the Ministry of Internal Affairs and Communications in 2016, over 90% of people ages 13 to 60 years old use the Internet in Japan [1]. IA has been found to be uncontrollable and damaging with excessive use of this technology [2]. Previous reports have shown that IA is one of the “impulse-control disorders” [3]. In 2013, the diagnosis of “Internet gaming disorder” was categorized as one of the problematic addictive behaviors in the Diagnostic and Statistical Manual of Mental Disorders, Fifth Edition (DSM-5) [4]. Furthermore, it has been argued that “gaming disorder,” including Internet gaming, should be included as a new concept in the International Classification of Disease, Eleventh edition (ICD-11) [5]. Thus, IA will be globally considered a disorder in the near future. It is important to prevent it from becoming a problem of the new era.

Recently, the Ministry of Education, Culture, Sports, Science and Technology—Japan (MEXT) has reported that many teachers and staff members working at schools had stress and poor mental health; therefore, the number of their school workers with mental illness had increased. One of the related symptoms of poor mental health is burnout syndrome (BOS). School teachers with BOS tend to have many causes; for example, teachers are asked to take on extra work, work at a higher quality, and spend an excessive amount of time on work. They work independently in spite of problems and find it hard to receive supportive feedback for their efforts [6]. As a result, it is well known that teachers experience burnout more easily. Maslach et al. developed the Maslach Burnout Inventory (MBI) [7] and defined BOS with three sub-concepts: “emotional exhaustion,” “depersonalization,” and “personal accomplishment” that can occur among individuals who work with people in some capacity. MBI was used for many studies all over the world after being issued [8, 9]. It has been contributed to the burnout research, allowing to be modified by researchers. In Japan, Kubo and Tao made the Japanese Burnout Scale (JBS) based on the MBI [10].

In a previous study about IA and BOS, it found a relationship between IA and BOS with Avci and Sahin reporting that BOS has a positive correlation with IA using an Internet addiction scale in Turkey among university healthcare staff members [11]. Another paper of adolescents reported that BOS indicates excessive Internet use, as well as BOS being caused by excessive Internet use [12]. Inaba et al. published a study of medical students in which the BOS group had a higher risk of IA by K-scale than the non-BOS group [13, 14].

MEXT has promoted incorporating Information and Communications Technology (ICT) in schools and improving the ICT environment at school. This policy has a purpose not only to educate teachers about how to create more attractive lessons and give them more knowledge for ICT utilization among students, but also to reduce the work burden among teachers with effective ICT [15]. Teachers will have increased opportunities to use the Internet in the future. Previous studies have indicated long time use of the Internet caused IA. [16]. A previous epidemiological study showed that at-risk Internet users were approximately 5% among high school teachers in a rural prefecture of Japan [17]. We are afraid that IA will increase among teachers in the near future. However, there have been few studies about the relationship of at-risk IA and BOS among teachers on a nationwide scale.

This study aims to research the relationship between at-risk IA and the Internet usage or BOS by conducting a nationwide cross-sectional survey and examining the factors associated with IA. Our findings will contribute to improving school mental health, including Internet addiction, among teachers.

## Methods

### Study design, participants, and procedures

This study was a cross-sectional survey by anonymous questionnaire. We used the single-stage cluster sampling methodology. The cluster unit of the sampling was schools. In 2015, there were 10,484 junior high schools in Japan [18]. We randomly selected 140 junior high schools (selection rate: 1.3%) based on student’s population using the National School Directory. All 3326 teachers enrolled in the sampled schools were subjects of this study. This survey was carried out in August and September 2016. We sent the study information and questionnaires to selected junior high schools and asked all teachers to participate in the study in the schools whose administrators consented to participation. They were informed that the survey was voluntary and confidentiality was ensured. Each school teacher sealed their completed anonymous questionnaire in the envelope, and administrators collected and returned them to us unopened. A completed questionnaire was considered evidence of the consent to the study. Responses were obtained from 73 junior high schools (response rate 52.1% in target schools). The responded schools had the following characteristics: 66 public schools vs. 7 private schools, 36 schools with 40 and less teachers vs. 37 schools with over 40 teachers, and 42 schools in rural areas vs. 31 schools in urban areas with over 5 million residents in each prefecture. Of all the 2080 responses, 384 did not have the complete data needed for the analysis. Thus, 1696 eligible respondents (eligible response rate 51.0% in target teachers) participated in this study.

### Measure

The questionnaire consisted of mainly three parts: Internet usage, at-risk IA by Internet Addiction Test (IAT), and BOS by Japanese Burnout Scale (JBS). The first part included biographical and background information, such as age, gender, position at their school and duration of service for school personnel, devices used for Internet access in the last 90 days, activities on the Internet in the last 90 days (communication, gaming, hobbies or entertainment, information gathering, shopping, investment or gambling, surfing the Internet, and others), and average time spent on the Internet per day in the last 90 days (both weekday use for work and weekend use for work and private purposes). The second part of the questionnaire asked participants about IA or at-risk IA, which was defined using the IAT. The third part of the questionnaire asked participants about BOS, which was defined using the JBS that has been used in many studies in Japan [19–21].

The IAT is a self-report measure comprised of 20 items rated on a 5-point Likert scale from “not at all” (1) to “very” (5). The total score on the IAT ranges from 20 to 100. Factor analysis of the IAT revealed six factors: salience, excessive use, neglecting work, anticipation, lack of control, and neglecting social life. These factors showed good to moderate internal consistency (*α* coefficient = 0.54–0.82) and concurrent validity, with salience being the most reliable [22]. The IAT has been the most commonly used measure in previous studies of IA [23–25]. Regarding the internal consistency of the Japanese version of IAT, it was reported that *α* coefficient = 0.93 [26]. This also shows the adequacy of using the total score of the Japanese version of IAT [27]. The total score defined IA or at-risk IA as follows: non-IA (total score< 40), at-risk IA (total score of 40–59), and IA (total score ≧ 60) [28].

The JBS consists of three factors: emotional exhaustion, depersonalization, and personal accomplishment. The MBI, which is the origin of JBS and shows BOS condition, was also separated into same three subscales [7]. Emotional exhaustion assesses feelings of being emotionally overextended and exhausted by one’s work. Depersonalization assesses an unfeeling and impersonal response toward recipients of one’s service, care, treatment, or instruction. “Personal accomplishment” assesses feelings of competence and successful achievement in one’s work with people. The JBS is a self-report measure comprised of 17 items: emotional exhaustion 5 items, depersonalization 6 items, and personal accomplishment 6 items, which are rated on a 5-point Likert scale from “not at all” (1) to “very” (5). The score of each factor ranges from 1 to 5, with the higher score indicating a stronger property of each factor. However, the items of personal accomplishment were inversion items, e.g., higher points of personal accomplishment mean better conditions. Thus, we replaced higher scores of personal accomplishment to lower scores of “decline of personal accomplishment” inversely in this study. We used consecutive scores by each factor in the JBS as each subscale scores of BOS [19, 20].

### Statistical analyses

#### Internet usage of junior high school teachers

There was no Internet addiction among 1696 participants of this study who had more than 70 points on the IAT. Therefore, we allocated the participants into either the at-risk IA group or the non-IA group. The Mann-Whitney test and the chi-square test were used to compare proportions between non-IA and at-risk IA. Student’s *t* test was used to compare IAT scores and JBS scores between the non-IA and at-risk IA.

#### Comparison of IA scores depending on degree of BOS

Scores of each factor of BOS were divided into quartiles. To compare the mean scores of the IAT in each quartile, analysis of variance was used. Analysis of covariance was used to adjust by age, device for Internet access (smartphone), Internet activity, hobbies or entertainment, shopping, surfing the Internet, gaming, and time spent on Internet access for work and private purposes on both weekdays and weekends.

#### Multivariate regression analysis

Multiple logistic regression analysis was used to assess odds ratio of each independent variable to the at-risk IA group. The following variables were used as independent variables in this analysis: age, device for Internet access (smartphone), time spent on Internet access for work and private purposes on both weekdays and weekends, activity on Internet (hobbies or entertainment, shopping, surfing the Internet, gaming), and each quartile category divided by subscales scores of JBS, because these variables had significant differences between non-IA and at-risk IA groups by Mann-Whitney test, chi-square, or Student’s *t* test.

The Statistical Package for the Social Science (SPSS Japan Inc., Version 24.0, Tokyo, Japan) was used for the analyses. All probability values were two-tailed, and all confidence intervals were estimated at the 95% level.

## Results

In our study, the at-risk IA group had 96 respondents (5.7%) and the non-IA group had 1600 respondents (94.3%). Table 1 shows the characteristics of respondents between the at-risk IA group and non-IA group. The at-risk IA group was younger and had a shorter length of service in school than those in the non-IA group. Gender and position at school had no relationship. In the BOS score, emotional exhaustion and depersonalization had higher scores in the at-risk IA group than in the non-IA group. However, decline of personal accomplishment had a lower score in the at-risk IA group than in the non-IA group.

**Table 1:** Characteristics of respondents or burnout score with at-risk Internet addiction(IA) and non-IA among junior high school teachers

Variables	Non-IA	At-risk IA	*P* value
	(*n* = 1600)	(*n* = 96)	
Age (years)^†^, *n*(%)			
< 30	312 (19.50)	42 (43.75)	< 0.001
30–39	363 (22.69)	18 (18.75)	
40–49	364 (22.75)	18 (18.75)	
50 <	561 (35.06)	18 (18.75)	
Gender^‡^, *n*(%)			
Male	942 (58.88)	62 (64.58)	0.269
Female	658 (41.13)	34 (35.42)	
Position at school^‡^, *n*(%)			
Administrator	129 (8.06)	5 (5.21)	0.386
Senior teacher	60 (3.75)	3 (3.13)	
Teacher	1160 (72.50)	67 (69.79)	
Full-time lecturer	131 (8.19)	11 (11.46)	
Nursing teacher	60 (3.75)	3 (3.13)	
Non-permanent teacher	60 (3.75)	7 (7.29)	
Duration of service (year)^†^, *n* (%)			
< 5	276 (17.25)	37 (38.54)	< 0.001
5–9	258 (16.13)	16 (16.67)	
10–19	328 (20.50)	14 (14.58)	
20–29	378 (23.63)	17 (17.71)	
30 ≦	360 (22.50)	12 (12.50)	
Burnout^§^, mean ± SD			
Emotional exhaustion	2.54 ± 0.88	3.04 ± 0.93	< 0.001
Depersonalization	1.67 ± 0.62	2.10 ± 0.75	< 0.001
Decline of personal accomplishment	3.35 ± 0.71	3.15 ± 0.66	0.009

Each number of the category variable shows the number of people, and each inside the parentheses shows % in each column

Burnout scale indicates mean ± SD

^†^Mann-Whitney test

^‡^*χ*^2^ test

^§^*t* test

Table 2 shows comparisons of Internet usage between the at-risk IA group and the non-IA group. Mean IAT scores were 46.68 ± 6.23 in the at-risk IA group and 25.51 ± 4.82 in the non-IA group. The at-risk IA group had a higher prevalence of having a smartphone. The at-risk IA group used the Internet more for gaming, hobbies or entertainment, shopping, and surfing the Internet than the non-IA group. The at-risk IA group spent a longer time on the Internet regardless of day of the week or purpose than the non-IA group.

**Table 2:** Comparisons of internet usage between at-risk IA and non-IA

Variable	Non-IA	At-risk IA	*P* value
	(*n* = 1600)	(*n* = 96)	
IAT score, Mean ± SD	25.51 ± 4.82	46.68 ± 6.23	
Device for Internet access ^‡^, *n*(%)^※^			
Smartphone	1226 (76.63)	84 (87.50)	0.014
Laptop computer	1337 (83.56)	78 (81.25)	0.554
Desktop computer	648 (40.50)	45 (46.88)	0.217
Tablet	524 (32.75	36 (37.50)	0.336
Feature phone	193 (12.06)	9 (9.38)	0.430
Others	17 (1.06)	4 (4.17)	
Number of devices^§^, mean ± SD	2.47 ± 0.86	2.67 ± 0.87	0.026
Activity on Internet^‡^, *n*(%)^※^			
Hobbies or entertainment	1078 (67.38)	85 (88.54)	< 0.001
Information gathering(work)	1389 (86.81)	82 (85.42)	0.695
Information gathering(private)	1280 (80.00)	78 (81.30)	0.766
Communication(private)	1141 (71.31)	77 (80.21)	0.060
Communication(work)	1093 (68.31)	71 (73.96)	0.247
Shopping	802 (50.13)	66 (68.75)	< 0.001
Surfing the Internet	416 (26.00)	56 (58.33)	< 0.001
Gaming	308 (19.25)	44 (45.83)	< 0.001
Investment or gambling	41 (2.56)	4 (4.17)	0.342
Others	18 (1.13)	0 (0.00)	
Time spent on Internet access^†^			
Weekdays use for work			
Not used	17 (1.06)	0 (0.00)	< 0.001
< 60 min	1189 (74.31)	57 (59.38)	
60 min ≦	394 (24.63)	39 (40.63)	
Weekdays use for private			
Not used	33 (2.06)	0 (0.00)	< 0.001
< 60 min	1110 (69.38)	21 (21.88)	
60 min ≦	457 (28.56)	75 (78.13)	
Weekends use for work			
Not used	230 (14.38)	12 (12.50)	0.001
< 60 min	1125 (70.31)	52 (54.17)	
60 min ≦	245 (15.31)	32 (33.33)	
Weekends use for private			
Not used	36 (2.25)	0 (0.00)	< 0.001
< 60 min	922 (57.63)	12 (12.50)	
60 min ≦	642 (40.13)	84 (87.50)	

Each number of the category variable shows the number of people, and each inside the parentheses shows % in each column

^※^Include multiple choices

^†^Mann-Whitney test

^‡^*χ*^2^ test

^§^*t* test

Table 3 shows the means of IAT scores according to the quartiles of 3 factors of JBS. The emotional exhaustion and depersonalization were positively associated with IAT scores. However, the highest quartile of decline of personal accomplishment had the lowest IAT scores. There was a statistically significant relationship between the IAT scores and each factor of BOS.

**Table 3:** Internet Addiction Test scores by quartile of each factor of burnout

Factor of burnout		*n*	Mean	SD	One-way ANOVA	ANCOVA
					*P* value	*P* value
Emotional exhaustion						
Quartile 1	1.00–1.80	425	24.7	5.70	< 0.001	< 0.001
Quartile 2	1.81–2.40	458	26.2	5.97		
Quartile 3	2.41–3.20	455	27.4	6.84		
Quartile 4	3.21–5.00	358	28.8	8.58		
Depersonalization						
Quartile 1	1.00–1.17	469	24.5	4.87	< 0.001	< 0.001
Quartile 2	1.18–1.50	442	26.2	6.39		
Quartile 3	1.51–2.00	421	27.4	6.88		
Quartile 4	2.01–5.00	364	29.4	8.66		
Decline of personal accomplishment						
Quartile 1	1.00–2.83	347	27.1	7.35	< 0.001	0.020
Quartile 2	2.84–3.33	401	27.3	7.48		
Quartile 3	3.34–3.83	438	27.1	7.01		
Quartile 4	3.84–5.00	510	25.7	5.94		

Table 4 shows the odds ratio and 95% confidence interval of each risk factor with at-risk IA determined using multiple logistic regression analysis. Internet activity such as gaming and surfing the Internet, time spent on Internet access on both weekdays and weekends for private purposes for 1 h or more, and depersonalization were positively associated with at-risk IA. On the other hand, highest quartile for decline of personal accomplishment had a significantly lower odds ratio with at-risk IA.

**Table 4:** Multivariate odds ratio and 95% confidence intervals for at-risk IA group

Variables	0dds ratio	95% CI
Age		
50 <	1.00	
40–49	1.19	0.57–2.47
30–39	0.73	0.34–1.57
< 30	1.42	0.71–2.84
Device for Internet access		
Smartphone (no)	1.00	
Smartphone	0.84	0.41–1.75
Activity on Internet		
Hobbies or entertainment (no)	1.00	
Hobbies or entertainment (yes)	1.34	0.65–2.77
Shopping (no)	1.00	
Shopping (yes)	1.18	0.71–1.97
Surfing the Internet (no)	1.00	
Surfing the Internet (yes)	1.88	1.16–3.05
Gaming (no)	1.00	
Gaming (yes)	1.83	1.13–2.98
Time spent on Internet access		
Weekdays use for work 60 min >	1.00	
Weekdays use for work 60 min ≦	0.83	0.48–1.42
Weekdays use for private 60 min >	1.00	
Weekdays use for private 60 min ≦	3.30	1.69 — 6.44
Weekends use for work not used	1.00	
Weekends use for work 60 min >	0.68	0.33–1.40
Weekends use for work 60 min ≦	0.99	0.43–2.25
Weekends use for private 60 min >	1.00	
Weekends use for private 60 min ≦	2.71	1.19–6.18
Burnout		
Emotional exhaustion quartile 1	1.00	
Emotional exhaustion quartile 2	1.12	0.49–2.57
Emotional exhaustion quartile 3	1.07	0.47–2.44
Emotional exhaustion quartile 4	2.07	0.87–4.91
Depersonalization quartile 1	1.00	
Depersonalization quartile 2	3.21	1.31–7.87
Depersonalization quartile 3	3.29	1.29–8.36
Depersonalization quartile 4	6.04	2.36–15.47
Decline of personal accomplishment quartile 1	1.00	
Decline of personal accomplishment quartile 2	0.90	0.47–1.70
Decline of personal accomplishment quartile 3	0.85	0.45–1.60
Decline of personal accomplishment quartile 4	0.38	0.19–0.78

*CI* confidence interval

## Discussion

Our study proved that at-risk IA was associated with the use of the Internet for many hours both on weekdays and weekends in private, using gaming and surfing the Internet. Furthermore, our findings indicated significant relationships between at-risk IA and two factors of BOS: depersonalization and decline of personal accomplishment even adjusting for those variables. These findings were clarified as the current situation of school mental health among teachers by a nationwide epidemiological survey.

The relationship between long-time Internet use and IA has been clarified in previous studies [12, 19]. Especially, Internet use for private purpose is more risky, and playing games and surfing the Internet can lead to overuse of the Internet. Many studies have revealed that the gaming is significantly related to the IA [29], but, to our knowledge, the study that surfing the Internet is related to the IA could not be found. It is well known that long-term use of the Internet is an IA risk factor. Tsumura et.al reported in the study researched in rural areas in Japan that the at-risk IA group spent a long time on the Internet [17]. We investigated on a nationwide scale, not in one area; then, the same result was obtained. In the present study, the Internet usage for a long time privately both weekdays and weekends were of higher risk than using the Internet for less than 60 min. When surfing the Internet, people who enjoy surfing the Internet, using the Internet without purpose, may easily lead to long time use.

Nanda et al. reported that BOS caused some addictive behaviors [30]. There were some previous studies of the relationship between BOS and addiction among employees [7, 11, 31–33]. Of these studies, they suggested that there were those associations between BOS and alcohol dependence or substance abuse. We found that those with BOS may have behavioral dependence such as Internet usage. BOS may easily lead to addictive behavior.

In factors of BOS in the study by Avci and Sahin [11], depersonalization was positively related to IA, and “diminished self-accomplishment” was negatively related to IA. Diminished self-accomplishment has the same meaning as decline of personal accomplishment in our study, and these results were similar to our findings. These previous studies [12–14] clarified the relationship between BOS and IA among youths. We found these relationships among teachers by a nationwide random sample. We clarified the positive relationship with depersonalization of BOS and IA, even adjusting for characteristics and Internet usage. Our findings proved the relationship of depersonalization and IA by multivariate analysis.

Okayasu reviewed previous research of IA as a psychosocial consequence and risk factors involved [26]. He described the risk factors of IA stemming from feelings of loneliness, lack of social skills, and lack of social support. He indicated IA was one coping mechanism for psychosocial stress. Depersonalization has been defined as “an unfeeling and impersonal response toward recipients” [7]. It has been reported that people with depersonalization have weaker communication or social skills. Depersonalization is one of interpersonal relationship disorders, and it is easy to show escape from the reality or aggression [10] With regard to interpersonal relations, Internet gaming disorder (IGD) was associated with problems with peers, a higher prevalence of both being bullied and bullying others [28]. Our study also indicated the positive relationship between higher scores of depersonalization and at-risk IA. Our result showed a reasonable result similar to previous studies. If IA occurs when coping with psychosocial stress, teachers work hard and they become exhausted both mentally and physically. They are imprisoned in the world of the Internet in trying to escape from real problems, and as a result, they may feel troubled or not be able to build better relationships with their students and colleagues. On the other hand, the personality characteristics of at-risk IA respondents included impulsively approaching new stimuli while being emotionally unstable with higher anxiety levels, and tending to avoid communication with others in real society. Yen et al. suggest three mechanisms to account for the association between IA and psychiatric symptoms [34]. First, psychiatric symptoms may lead to the onset or persistence of IA. Second, IA may precipitate psychiatric symptoms. Third, IA and psychiatric symptoms may increase vulnerability to each other. Anxiety, depression, and attention-deficit hyperactivity disorder (ADHD) are known as psychiatric symptoms associated with IA [35–37]. We can easily guess that people with these symptoms tend to have depersonalization, and then they are not able to build good interpersonal relationships. Our results suggest that findings of early-stage depersonalization may prevent or screen IA among junior high school teachers. It is important to pay attention to teacher behavior and note changes toward depression or aloofness to students, parents, and colleagues, or a refusal to connect with co-workers and relations, instead devoting their time to using the Internet.

Sano et al. reported an association between decline of personal accomplishment and social activity disorder [38]. It indicates that decline of personal accomplishment is against IA. It seems that teachers with decline of personal accomplishment tend to reject contact with real society in addition to society via the Internet. Furthermore, it is said that decline of personal accomplishment is less related to a stress factor [21]. Kubo suggested that decline of personal accomplishment such as factors other than the stressor derived from the workplace environments, perhaps experiences and personal characteristics are involved in the decline of personal accomplishment feeling [19]. Teachers who cannot obtain support from other teachers have been reported to be prone to decline of personal accomplishment in Japan [19]. Previous studies have reported that there was a relationship between IGD and individual aspects of psycho-social tendencies, such as loneliness, low self-esteem, low self-efficacy, low life-satisfaction, and lower educational and career attainment [29]. Although we cannot clarify the causal relationship in this study, not only the workplace environment but also the individual characteristics of teachers may cause burnouts and depend on the Internet world. Our study found a highest quartile for decline of personal accomplishment had lower odds ratio with at-risk IA. Thus, we guess that teachers with at-risk IA may have higher personal accomplishments in the Internet world.

This study has several limitations. First, our study used a cross-sectional design, which does not prove a causal relationship between BOS and IA. Second, since there is no consensus on the formal diagnostic criteria and gold standard measures for IA, the diagnostic accuracy properties of the IAT cut-off scores are yet to be established. Third, we used the JBS that was developed based on the MBI using situations of working people in human service occupations, and it was used in many studies in Japan. However, it was difficult to compare this study in detail with worldwide studies. Fourth, this study was a national survey, but we obtained a low response rate. We need to conduct a study with a higher response rate. Furthermore, we did not pose questions which vary depending on the work of the junior high school teachers, and these items may influence BOS. Then, we could not distinguish between personal accomplishment in the real world and in the Internet world. Since there may be differences between males and females in how to use the Internet, it should be examined in detail by gender. Finally, our results may be underestimated as we cannot rule out the possibility that there was some rejection to reply to the questionnaire as a result of heavy IA or BOS.

## Conclusion

Our study proved that at-risk IA was associated with using the Internet for many hours for private use, using gaming and surfing the Internet. Our results suggest that teachers should be instructed to use the Internet for private use properly. This study clarified the relationship between at-risk IA and BOS among junior high school teachers by a nationwide epidemiological survey. Our findings indicated a significant relationship between BOS and at-risk IA even adjusting for those variables. Of the three factors of BOS, depersonalization had a positive relationship with at-risk IA. Therefore, our results suggest that findings of early stages of depersonalization may prevent at-risk IA among junior high school teachers. Those who have at-risk IA may feel personal accomplishments through use of the Internet. Our findings will contribute to improving school mental health among teachers.

## Abbreviations

ADHD: Attention-deficit hyperactivity disorder
BOS: Burnout syndrome
DSM-5: Diagnostic and Statistical Manual of Mental Disorders, Fifth Edition
IA: Internet addiction
IAT: Internet Addiction Test
ICD-11: International Classification of Disease, Eleventh edition
ICT: Information and Communications Technology
IGD: Internet gaming disorder
JBS: Japanese Burnout Scale
MBI: Maslach Burnout Inventory
MEXT: Ministry of Education, Culture, Sports, Science and Technology—Japan
